# Beta and gamma oscillatory activities associated with olfactory memory tasks: different rhythms for different functional networks?

**DOI:** 10.3389/fnbeh.2014.00218

**Published:** 2014-06-23

**Authors:** Claire Martin, Nadine Ravel

**Affiliations:** ^1^Laboratory Imagerie et Modélisation en Neurobiologie et Cancérologie, CNRS UMR 8165, Université Paris Sud, Université Paris DiderotOrsay, France; ^2^Team “Olfaction: Du codage à la mémoire,” Centre de Recherche en Neurosciences de Lyon CNRS UMR 5292, INSERM U1028, Université Lyon 1Lyon, France

**Keywords:** beta and gamma oscillations, odor learning, behavior, olfactory bulb, piriform cortex

## Abstract

Olfactory processing in behaving animals, even at early stages, is inextricable from top down influences associated with odor perception. The anatomy of the olfactory network (olfactory bulb, piriform, and entorhinal cortices) and its unique direct access to the limbic system makes it particularly attractive to study how sensory processing could be modulated by learning and memory. Moreover, olfactory structures have been early reported to exhibit oscillatory population activities easy to capture through local field potential recordings. An attractive hypothesis is that neuronal oscillations would serve to “bind” distant structures to reach a unified and coherent perception. In relation to this hypothesis, we will assess the functional relevance of different types of oscillatory activity observed in the olfactory system of behaving animals. This review will focus primarily on two types of oscillatory activities: beta (15–40 Hz) and gamma (60–100 Hz). While gamma oscillations are dominant in the olfactory system in the absence of odorant, both beta and gamma rhythms have been reported to be modulated depending on the nature of the olfactory task. Studies from the authors of the present review and other groups brought evidence for a link between these oscillations and behavioral changes induced by olfactory learning. However, differences in studies led to divergent interpretations concerning the respective role of these oscillations in olfactory processing. Based on a critical reexamination of those data, we propose hypotheses on the functional involvement of beta and gamma oscillations for odor perception and memory.

## Introduction

Among the functional particularities of the olfactory system, we wish to stress its privileged access to limbic structures and its predisposition to rhythmicity. In the absence of thalamic relay, the olfactory receptors are only two and three synapses distant from the cortical amygdala and the hippocampus respectively. This singularity could partly explain why olfactory experience has been reported to be so efficient to shape odor representations (Wilson and Stevenson, 2003; Davis, 2004). In adults, anatomic and functional plasticity related to odor learning occur at every step of the olfactory system. As early as in the olfactory mucosa, olfactory learning increases the number of sensory neurons specific to a trained odorant (Jones et al., 2008; Dias and Ressler, 2014). Studies carried out in the main olfactory bulb (MOB) and the piriform cortex (PCx) reported long-lasting modifications of neuronal activity and synaptic efficiency in various learning contexts (Barkai and Saar, 2001; Mouly et al., 2001; Mouly and Gervais, 2002; Martin et al., 2004b; Sevelinges et al., 2004; Mandairon and Linster, 2009; Restrepo et al., 2009; Wilson and Sullivan, 2011; Royet et al., 2013).

The olfactory system is also highly dynamic. On the one hand, odorant detection and coding are constrained by respiratory modulation through breathing. The sniff cycle controls the firing pattern of olfactory neurons in time and is suggested to be the functional time unit for odor processing (Buonviso et al., 2006; Kepecs et al., 2006; Wachowiak, 2011). On the other hand, odor processing has been associated with oscillations of the local field potential (LFP) both in insects (Perez-Orive et al., 2002) and mammals (Kay et al., 2009). Those signals reflect a weighted average of synchronized dendro-somatic components of neuronal processing within a neural population (Buzsáki et al., 2012). Because they underlie coincident activity, oscillations would favor temporal coordination of sensory information within brain areas and facilitation of its transfer across regions (Varela et al., 2001; Siegel et al., 2012). Accordingly, they are ideally suited to subserve memory processes such as encoding, consolidation and retrieval (Engel et al., 2001; Varela et al., 2001; Tallon-Baudry et al., 2004; Fell and Axmacher, 2011).

The present review will leave apart the respiratory modulation which has already been the object of several recent reviews (i.e., Buonviso et al., 2006; Kepecs et al., 2006; Scott, 2006; Wachowiak, 2011). The aim here is to synthesize our current knowledge about the conditions in which the other two main oscillatory rhythms linked to odor processing, namely beta (15–40 Hz) and gamma (60–90 Hz), are observed at the first stages of olfactory processing, the MOB and the PCx. A majority of studies designed to decipher odor coding have been performed in anesthetized animals. These studies have been essential for understanding the activity of single neurons in response to odorants both in the MOB and the PCx (Buonviso et al., 2003; Fletcher and Wilson, 2003; Litaudon et al., 2003; Chapuis and Wilson, 2012; Fukunaga et al., 2012). In the present review, we will focus exclusively on LFP recordings performed in awake, behaving animals. One benefit of chronic LFP recordings as compared to single unit recordings is the ability to follow the evolution of rhythmic activities across cerebral areas throughout training as a means of tracking learning-related changes. Because oscillatory activities are transient, their detection and precise description requires operant devices, in which the timing of odorant onset and offset can be precisely controlled. Comparing the relation between behavior and LFP oscillations in various conditions, we came to propose hypotheses on the functional involvement of beta and gamma oscillations in the context of odor processing. Far to be exhaustive, the scope of this review is to consider the respective putative role of these two oscillatory rhythms in odor coding and memory.

## The olfactory system and its oscillations

More than any other sensory system the olfactory system has early been reported to be oscillatory (Adrian, 1942; Freeman and Schneider, 1982; Gray, 1994). This specificity is most probably due to two parameters, the nature of the stimulus and the organization of olfactory areas. First, odorant molecules are slow to reach the detector, compared to sound or light. They travel with nasal airflow, and do not reach simultaneously the different parts of the nasal cavity. Because odorant onset cannot be sharp, it most often fails to elicit evoked potential. Second, as it will be described below, the central olfactory relays (MOB and PCx) are tightly interconnected and host specific features; the mitral-granule dendrodendritic reciprocal synapses in the MOB, and a dense network of associational fibers in the PCx.

The olfactory sensory neurons present in the nasal cavity are the point where the odorant chemical information is transduced and transmitted to the brain (Zufall and Munger, 2001). All the olfactory sensory neurons that express the same molecular receptor converge onto a few glomeruli, well identified microdomains containing the first synapse of the olfactory information path (Zou et al., 2009). In the absence of thalamic relay, the MOB has been considered already as an associative structure where inhibition plays a major role. Olfactory signal that travels in the principal excitatory neurons, mitral and tufted (MT) cells, is gated at two levels within the structure. Surrounding glomeruli, juxtaglomerular cells include astrocytes and various types of neurons: excitatory external tufted cells, periglomerular cells and short axon cells. While periglomerular are GABAergic cells, short axon cells, which processes extend across several glomeruli, have two opposite actions by releasing both GABA and Dopamine (Liu et al., 2013). Deeper in the structure, the specific interaction between granules and MT cells via dendrodendritic reciprocal synapses is a key element for the large oscillatory activity displayed in the olfactory system. MT cells axons coalesce into the lateral olfactory tract and project to numerous areas termed as the olfactory cortex. Privileged targets of the MOB are the anterior olfactory nucleus and the anterior PCx (Haberly, 2001; Cleland and Linster, 2003; Hintiryan et al., 2012). MT cells also contact in a lesser extent, the posterior PCx, the lateral entorhinal cortex, the olfactory tubercle, the ventral tenia tecta and the anterior cortical complex of the amygdala.

The PCx is anatomically and functionally divided into two parts: a rostral region (anterior PCx) mostly connected to the other olfactory areas and a caudal region (posterior PCx) in connection with higher cognitive regions and characterized by dense associational connectivity (Haberly, 2001; Litaudon et al., 2003; Bekkers and Suzuki, 2013). Indeed, the anterior part of the PCx has strong bidirectional projections to the posterior part of the structure and to the MOB and the anterior olfactory nucleus. On the contrary, the posterior PCx has dense feedforward projections to numerous cortical and subcortical regions including high-order association areas, but lacks functional projections to the anterior PCx. In addition, feedback projections from the posterior PCx to the MOB are sparse. A noticeable point in the functional anatomy of the PCx is the presence of abundant associational connections, sparser in the anterior than in the posterior PCx (Hagiwara et al., 2012). Anatomo-functional connectivity of the PCx already suggests a key role of this structure in the elaboration of complex mechanisms of olfactory perception and memory (Gottfried, 2010; Wilson and Sullivan, 2011). Differences between anterior and posterior PCx would sustain complementary memory processes: as suggested in the literature, the anterior PCx would mediate odors matching such as generalization, discrimination or pattern completion (Wilson and Stevenson, 2003; Chapuis and Wilson, 2012) whereas the posterior PCx would rather link odor to previously learned non-olfactory information (Haberly, 2001).

The MOB and the PCx are densely interconnected. The lateral olfactory tract carries odor information from the MT cells to pyramidal cells. In turn, pyramidal cells send axon collaterals to the MOB. These glutamatergic fibers synapse almost exclusively on the different type of inhibitory interneurons contained in the MOB. They have a major inhibitory effect on the structure at two levels: the glomerulus via periglomerular cells, and the mitral cell via granule cells. Interestingly, the strongest drive is to deep and superficial short axon cells, the main source of inhibition onto granule and periglomerular cells (Boyd et al., 2012). Centrifugal projections to the MOB do not only originate from the PCx (Matsutani, 2010). Moreover, acetylcholine, norepinephrine, and serotonin projections modulate the activity of the MOB and the PCx (Linster and Hasselmo, 2001; Ennis et al., 2007; Rothermel et al., 2014). They can inhibit as well as disinhibit glomerular activity and MT cells.

Dense interconnections between and within olfactory structures are conducive to the emergence of oscillations. Presumably for this reason, the MOB and the PCx have been very early seen as good models to study rhythmic activities in the brain, and have been the target of pioneering electrical recording of brain oscillations (Adrian, 1942, 1950). Few years later, Lavin et al. (1959) performed the first recording of the electrical activity of the MOB in awake, unrestrained cats chronically implanted with electrodes. They reported bursts of activity related to the arousal of the animal. Since that time, numerous studies have recorded intracerebral LFPs in the MOB and the PCx in behaving animals. In these brain areas, even raw signals overtly display different types of oscillations that can be easily defined in sub-classes according to their frequency range and to the moment they occur in relation with external events.

**Figure 1:**
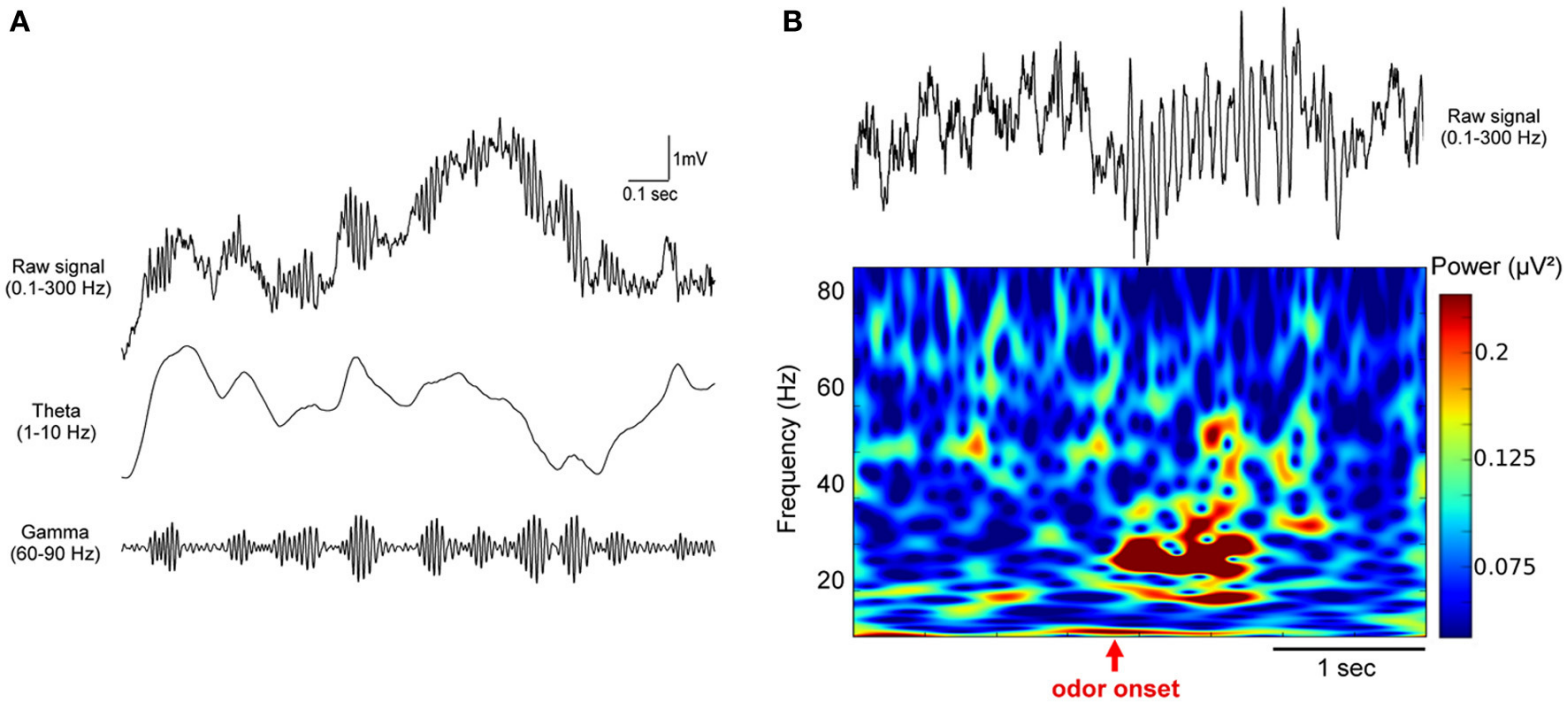
**Odor stimulation modifies beta (15–35 Hz) and gamma (60–90 Hz) oscillations in the olfactory bulb**. Example of LFP traces recorded in the olfactory bulb in freely moving mice. **(A)** Raw LFP signal (0.1–300 Hz) on first row is filtered in the theta (1–10 Hz) and the gamma (60–90 Hz) bands, showing the close relation between gamma bursts and the respiratory modulation. **(B)** Raw LFP signal (0.1–300 Hz) and corresponding time-frequency power representation in a mouse conditioned in a Go/No-Go task. Time-frequency plot was obtained based on Morlet wavelet analysis. It represents the power of the signal (as indicated by the color scale) as a function of time (x-axis) for each frequency (y-axis). Odorant onset is indicated by the vertical red arrow. Note that odor elicits an overall decrease in the gamma band and an increase in the beta band power.

Oscillatory activities in the olfactory system covers a broad frequency band comprised between 1 and 150 Hz. In the MOB and the PCx, three rhythms dominate. The larger and most obvious is linked to the respiration and occurs in a frequency range overlapping with the hippocampal theta rhythm (~1–10 Hz) (Kay et al., 2009). In awake and motivated animals, regular bursts of fast oscillations, i.e., the gamma rhythm (~60–90 Hz) are nested onto the respiratory modulation, occurring at the transition between inspiration and expiration (Buonviso et al., 2006; Manabe and Mori, 2013) (for an example see Figures 1A, 3A). Odorant presentation most often elicits beta oscillations (~15–40 Hz) of variable amplitude, but has also been associated with gamma increase restrained to the MOB (Beshel et al., 2007). Finally, in the MOB, some sporadic long lasting bursts of low frequency gamma (~35–65 Hz) can occur during exploration (Kay, 2003). The boundaries of these rhythms are sometimes variable in frequency, depending on the animal species; as a consequence we will consider their functional condition of emergence rather than their absolute frequency.

## Odor-evoked modulation of LFPs, influences on beta and gamma band oscillations

The rhythm classically studied in the olfactory system has been the gamma band (~60–90 Hz). In the absence of imposed odorant stimulation, in particular when animals freely explore their environment, the presence of gamma bursts, regularly nested at the transition between inspiration and expiration characterizes the LFP in the MOB (Figure 1A) and at least in the anterior PCx. Beside the ubiquitous nature of gamma bursts in the olfactory system, the fact that gamma frequency has been recognized as the gold standard for sensory coding following the work of Wolf Singer in the visual system (Singer, 1993) probably drew the attention of the community on this frequency range. A detailed historical review about gamma oscillations in the olfactory system can be found in Rojas-Líbano and Kay (2008).

Gamma oscillations have been extensively analyzed in several regions of the olfactory system by Walter Freeman (Freeman, 1960; Freeman and Schneider, 1982; Eeckman and Freeman, 1990), who focused analyses on a rather large frequency range (20–90 Hz). As it has been reported in numerous studies, gamma bursts amplitude increases mostly when the animal is in an attentive state (Bressler, 1984; Eeckman and Freeman, 1990). This relationship to attention and motivation is easy to observe in the initial phase of any training. Indeed, our data suggest that when a rat is placed in a novel environment, the amplitude of spontaneous activity related gamma bursts increases very rapidly as the animal becomes more familiar with the arena and aware of what is going to happen (Martin et al., unpublished data). By recording EEG using a 64-electrodes grid at the surface of the MOB of small mammals, Freeman analyzed the spatial distribution of odor-induced gamma bursts amplitude, considering the brain as a chaotic system. In two of the most famous papers (Freeman and Schneider, 1982; Di Prisco and Freeman, 1985), authors examined this pattern during and after either an aversive or appetitive odor conditioning. They reported that spatio-temporal motifs emerging from gamma bursts analysis were relatively independent of odor presentation and more related to the significance of the odor. They proposed that gamma oscillatory activity modulations were mostly related to the context and expectations of animals. These studies were pioneering in considering the MOB as a central element of a broader network underlying odor representations, rather than a passive odor relay. They were also the first evidence that odor processing at this early sensory stage already takes into account the context and the experience of the animal. Interestingly, as reported in several articles from the same group (Di Prisco and Freeman, 1985), the fact that spatial distribution of iso-amplitude gamma bursts is indeed modified by experience does not mean that odorant presentation increases gamma oscillations amplitude. On the contrary, the mean gamma power over the MOB remained stable or decreased by 15–35% during odorant sampling. In a series of experiments we performed at the beginning of the 2000's we observed that odorant sampling in the context of a Go/No-Go task was first associated with a strong and transient decrease of gamma oscillatory activities at the onset of odor. This power decrease was observed in naïve animals and was amplified as rats became experts for the odorants used in the task. Gamma depression was transient and most often followed by a light rebound effect before a return to baseline activity with calibrated regular gamma bursts nested on a slower respiration-related activity (Ravel et al., 2003; Martin et al., 2004b, 2006).

The decrease in gamma activity is most often replaced by the emergence of an activity in the beta band (15–40 Hz, centered around 25 Hz) that is never observed in the absence of odorant in normal condition (Figure 1B). This shift in the oscillatory dynamics between gamma and beta frequencies is characteristic of odorant sampling in awake animals and have been reported in numerous studies in the MOB (Gray and Skinner, 1988b; Martin et al., 2004b; Lowry and Kay, 2007; Lepousez and Lledo, 2013; Chery et al., 2014) and the PCx (Martin et al., 2006). Interestingly, beta oscillations elicited by odorant stimulations have been characterized for the first time in the dentate gyrus of the hippocampus where inhalation of toluene by the rat produced fast-wave bursts (Vanderwolf, 1992). This group conducted many studies in awake rats submitted to passive presentations of odorants that were supposed to be innately relevant or naturally aversive to the animals (urine, feces, toluene, predator odors…) (Vanderwolf, 1992; Heale and Vanderwolf, 1994; Zibrowski and Vanderwolf, 1997; Chapman et al., 1998; Zibrowski et al., 1998; Vanderwolf and Zibrowski, 2001; Vanderwolf et al., 2002). They showed that a low frequency wave (around 20 Hz) was elicited by these odorants in a large network covering the MOB, the PCx, and limbic structures (entorhinal cortex, dentate gyrus). They also observed in the PCx that the repeated presentation of odorants (10–15 trials) leads to a gradual enhancement of beta wave amplitude that persists for several days (Vanderwolf and Zibrowski, 2001). In a subsequent study, Lowry and Kay (2007) have also found large beta activity during passive presentation of some specific odorants. However, they reported that all the odorants that showed significantly higher beta power were in a certain range of vapor pressure, between 1 and 120 mmHg. Interestingly, this range includes TMT, a component of fox feces, and toluene. In urethane anesthetized rats, similar observations were made that the molecular feature of odorants influenced the probability of emergence of beta oscillations (Cenier et al., 2008). Consequently, the reason why some odorants elicited higher beta power could be due to their volatility rather than their innate value. However these studies have been conducted using cotton swab presentations, a condition in which the odorant concentration and duration is more difficult to calibrate. Elevated odorant concentration may by itself be fearful and/or aversive for macrosmatic animals such as rodents. Indeed, in both sets of data, beta power enhancement induced by repeated exposure to the same series of odorants suggests that other processes than pure olfactory detection occur like some odor recognition and classification. Oscillatory activities related to naturally meaningful odorant molecules have also been found in the accessory olfactory bulb, which receives its sensory input from the vomeronasal organ. In awake female mice, male urine exposure significantly increases LFP power in frequencies overlapping with beta rhythm (ranging from 8 to 24 Hz) at the vicinity of the MT cells layer. Interestingly, following mating, the power of the LFP oscillations recorded under baseline conditions is dramatically increased across all frequency bands, suggesting that some form of synaptic plasticity has occurred (Binns and Brennan, 2005).

In conclusion, as presented in Table 1, most of the studies using odorant presentation have shown that they elicits a shift in frequency for the oscillatory activities recorded in the MOB and the PCx. Respiration locked gamma band activity (60–90 Hz) decreases and a slower beta oscillation (15–40 Hz) emerges. The reason why the pioneering studies led by Walter Freeman did not described such a systematic shift could be explained by the fact that recording were not performed in deep layers but at the surface of the cortex (Buffalo et al., 2011).

**Table 1:** **Beta oscillations recorded during odor presentation in awake mammals**.

**Study**	**Species**	**Structures**	**Frequency (Hz)**	**Context**
Gray and Skinner, 1988a,b	Rabbit	Olfactory bulb	15–25	Repeated presentation of unreinforced odors
Boeijinga and Lopes da Silva, 1989	Cat	Olfactory bulb, posterior piriform cortex, entorhinal cortex	~18	Exploratory sniffing, Go/No-Go-like with male and female urine
Dumenko, 1995	Dog	Cortical areas including the olfactory bulb	9–20	Association between odor and food dispenser
Heale and Vanderwolf, 1994; Zibrowski and Vanderwolf, 1997; Zibrowski et al., 1998; Vanderwolf and Zibrowski, 2001; Vanderwolf et al., 2002	Rat (and vole)	Piriform cortex and dentate gyrus	~20	Passive odor presentation
Chapman et al., 1998	Rat	Olfactory bulb, piriform cortex, entorhinal cortex, dentate gyrus	15–35	Passive odor presentation
Kay and Freeman, 1998	Rat	Olfactory bulb, piriform cortex, entorhinal cortex, dentate gyrus	12–35	Odor discrimination (Go/No-Go) with fixed inter trial interval
Chabaud et al., 2000	Rat	Olfactory bulb, piriform cortex, lateral entorhinal cortex	15–30	Exposure to behaviorally relevant odors
Ravel et al., 2003; Martin et al., 2004a,b, 2006; Gourévitch et al., 2010	Rat	Olfactory bulb, piriform cortex, entorhinal cortex, hippocampus	15–40	Odor discrimination (Go/No-Go)
Lowry and Kay, 2007	Rat	Olfactory bulb, anterior piriform cortex	15–30	Passive odor presentation
Hermer-Vazquez et al., 2007	Rat	Posterior piriform cortex, Motor area I, magnocellular red nucleus	13–30	Odor discrimination (Go/No-Go reach-to-grasp food task)
Fuentes et al., 2008	Rat	Olfactory bulb	10–40	Odor discrimination (2 alternative choice)
Chapuis et al., 2009	Rat	Olfactory bulb, piriform cortex, orbito-frontal cortex, basolateral amygdala, insular cortex, infralimbic cortex	15–40	Conditioned odor aversion
Kay and Beshel, 2010	Rat	Olfactory bulb, piriform cortex	15–35	Odor discrimination (2 alternative choice)
Carlson et al., 2014	Rat	Olfactory bulb, Olfactory tubercle	15–35	Passive odor presentation
Igarashi et al., 2014	Rat	Dentate gyrus, entorhinal cortex	20–40	Odor discrimination (Odor-place association)
Lepousez and Lledo, 2013	Mouse	Olfactory bulb	20–40	Odor discrimination (Go/No-Go)
Chery et al., 2014	Mouse	Olfactory bulb	15–35	Association between odor and food dispenser

## Network sustaining beta and gamma rhythms in the olfactory bulb

Is the same network involved in the generation of beta and gamma oscillations? As presented above, odorant presentation often leads to a gamma decrease coupled to a beta increase suggesting that the two rhythms share a common cellular substrate. If this is easily noticeable during odor-reward learning tasks, some studies involving passive and non-reinforced odor presentations find both gamma and beta enhancement during odor sampling (Lowry and Kay, 2007; Carlson et al., 2014). Stimulus delivery, not constraint by a nose poke may not be continuous, which could explain this discrepancy. Indeed, Lowry and Kay (2007) mention that within single investigation period, bursts at each frequency actually alternate, as it is reported in urethane anesthetized rats. In our hands, passive odor presentations induced the same shift from gamma to beta rhythm (Chabaud et al., 2000). The conditions of generation of gamma oscillations in the MOB have been extensively studied by computational modeling and electrophysiology *in vivo* or *in vitro*. Much less data have been collected concerning beta oscillations.

Gamma bursts present during spontaneous activity are generated in the MOB under the influence of spontaneous input from the neuroreceptors located in the nasal cavity (Hernandez-Peon et al., 1960; Gray and Skinner, 1988a), and are then transmitted to the PCx (Bressler, 1984; Mori et al., 2013). Indeed, blocking descending centrifugal influences by cooling or local infusion of anesthetic leads to an increase and not a decrease of MOB gamma bursts amplitude (Gray and Skinner, 1988a; Martin et al., 2006). In addition, the section of the lateral olfactory tract, which interrupts the transmission of the olfactory signal from the MOB to the PCx, selectively abolishes gamma bursts in the PCx (Neville and Haberly, 2003). On the contrary, pharmacological removal of centrifugal influences to the MOB abolishes beta oscillations in both the MOB and the PCx (Martin et al., 2006). Therefore, the major difference between the two rhythms is that gamma oscillations are generated locally within the MOB, whereas beta oscillations require intact bidirectional connectivity at least between the MOB and the PCx.

Within the MOB, gamma oscillations have been shown to be supported by the reciprocal synapse between mitral and granule cells (Nusser et al., 2001; Bathellier et al., 2006; Schoppa, 2006; Lagier et al., 2007; David et al., 2009). Computational models agree with the fact that these oscillations require an appropriate balance between excitation and inhibition, consistent with the mechanism proposed for gamma-band generation in other cortical areas (Cannon et al., 2014). Recent data have confirmed these mechanisms in awake mouse. They show that increasing the excitation/inhibition balance of MT cells via a decrease of GABAa receptors inhibition or local injection of glutamatergic agonists boosts gamma oscillatory power (Lepousez and Lledo, 2013). Consistently, selective MT cells drive using optogenetic technique causes a 5–10 fold increase of gamma oscillations without affecting other frequency bands. By scanning different frequency for light pulses (between 25 and 90 Hz), the authors show that the maximal response of the LFP occurs around 66 Hz, which corresponds to the dominant frequency of spontaneous gamma oscillations. Interestingly, the same GABAa receptors antagonist picrotoxin, which enhances gamma oscillations, leads to a reduction of beta oscillations power by more than 65%. On the contrary injection of MK801, an NMDA receptor antagonist, reduce gamma oscillations power without affecting beta oscillations (Lepousez and Lledo, 2013). Finally, modifications of beta and gamma oscillations observed in the presence of glutamate reuptake blockers argue for a role of glutamate spillover in constraining synaptic time constants. They suggest that MT cells glutamate release may locally change NMDA and AMPA mediated excitation (Martin et al., 2012; Lepousez and Lledo, 2013).

Taken together, these data imply that the two rhythms require the MOB network to exist. Even if they are both constrained by inhibition onto MT cells, this inhibition is likely to occur under different forms: either locally within the MOB network (granule and periglomerular cells) or remotely through centrifugal feedback. Because beta oscillations require intact connections between the MOB and at least the PCx, they are likely to emerge when the cerebral network engaged is broader. In the following part, we will examine which behavioral conditions are associated with either of the two oscillations, and how the distinction can have a functional readout in the context of learning.

## Odor learning induced modifications: different rhythms for different learning tasks?

As we discussed earlier, beta oscillatory activity has been observed in the olfactory system in naïve animals in response to toxic or aversive odorants (Zibrowski et al., 1998; Vanderwolf et al., 2002). When exposed to a neutral unfamiliar odorant, only weak beta oscillations are observed but their amplitude increases through training as soon as this odor starts to acquire a behavioral meaning for the animal (Ravel et al., 2003; Martin et al., 2004b). Such a learning-induced increase in beta power has been observed in several structures associated with odor processing (MOB, PCx, entorhinal cortex, and hippocampus) and for a variety of behavioral paradigms (see Table 1): Go/No-Go task (Ravel et al., 2003; Martin et al., 2004b, 2007; Gourévitch et al., 2010; Lepousez and Lledo, 2013), two-alternative choice task (Fuentes et al., 2008) and aversive learning (Chapuis et al., 2009). However, a few studies, with similar operant conditioning, report an odor evoked gamma increase instead of a change in beta activity (Beshel et al., 2007; Rosero and Aylwin, 2011).

It is easily arguable, when comparing the studies where LFPs have been recorded in olfactory structures in different operant tasks that the presence or not of substantial beta oscillations seems to be strongly dependent on the behavioral context of the task, and the cognitive strategy required to solve it (Kay et al., 2006). Go/No-Go and two-alternative choice have been the two main tools used to assess odor discrimination and learning in rodents. In the case of the Go/No-Go task, two odorants are delivered in a random order, one is positively rewarded (CS+; sucrose) and another is not rewarded or associated with a negative reinforcement (CS-; quinine). Initially, both odorants are neutral to the animals, and do not elicit any particular behavior. Over the course of training, animals learn to associate each odorant with the corresponding reward, and exhibit a differential behavior in response to the two odorant stimuli. Reaching the behavioral criterion for good performances takes several sessions, a duration that can vary with the difficulty of the task, which depends itself on the qualitative proximity of the odorants used.

In the Go/No-Go task, we have constantly found beta power increase during learning for both the CS+ and CS− (Martin et al., 2004a,b, 2006), raising the question of the link between this activity and the chemical feature of the odorant in one hand, or the odor meaning on the other hand. Oscillations in the olfactory system are triggered by odorant sampling and are likely to carry some aspects of odorants, as it has been demonstrated in anesthetized animals (Cenier et al., 2008). Indeed, by recording LFP signals from four different locations within the MOB, we showed that the main characteristics of beta oscillations (frequency and amplitude) are not homogeneous across the MOB, contrarily to gamma bursts recorded during spontaneous activity. Moreover, during learning, a stronger beta power is found in the posterior part of the structure (Martin et al., 2004b). Distinct odors evoke different amplitude levels of beta oscillations, irrespectively of the reward they are associated to. For a given animal, two different CS+ odorants can evoke distinct beta amplitude (Martin et al., 2004a), and a reversal procedure for an odorant pair (inverse learning contingencies) does not lead to the mirror image of the beta activity for each odor (Martin et al., 2007). Taken together, these data show that specificity of beta oscillations after learning would convey some feature of the odorant. However, it is likely that beta rhythm also reflects the odor signification acquired through learning.

Contrarily to the Go/No-Go, where only one odor is reinforced, the two-alternative choice task is symmetrically rewarded and a pellet is delivered for each correct response. This task, that seems more demanding for a rodent, is indeed often acquired slowly by the animals, and with a lower final performance (Friedrich, 2006; Slotnick, 2007). However, a recent study has shown that adjustment of parameters could allow to attain the same level of accuracy than for the Go/No-Go task in the same laps of time (Frederick et al., 2011). Besides the difficulty of the task, we can make the hypothesis that these two tasks involve different strategies and thus activate different brain circuits. In the two-alternative choice paradigm, odors can elicit high amplitude beta oscillations (10–30 Hz) and a significant decrease in the gamma band (70–100 Hz) (Fuentes et al., 2008). However, Beshel et al. (2007) using this task to compare successive odor pairs discriminations obtained different results. In this study, as expected, the animals are faster to reach the criterion for molecularly dissimilar odorants than for similar ones. Moreover, once animals are at the criterion for the discrimination, odor evoked gamma (60–85 Hz) power is very high for fine discriminations and almost absent for coarse ones. Within a given session, gamma power increases almost linearly across trial block but resets at the beginning of each session even if performances are improved. Interestingly, gamma increase is restricted to the MOB and does not propagate to the PCx. However, besides this gamma response, beta oscillations are also observed in three interconnected olfactory areas (MOB and anterior and posterior PCx) and only the beta band exhibits consistently elevated coherence levels between these three areas during odor sniffing across all odor pairs, classes (alcohols and ketones), and discrimination types (fine and coarse) (Kay and Beshel, 2010).

As mentioned earlier, the respiratory modulation influences odor processing. In anesthetized-tracheotomized animals, an airflow change is sufficient to change the relative power of beta and gamma frequency bursts (Courtiol et al., 2011). The direct relation between sniffing properties and oscillatory patterns during olfactory conditioning is still an open question in awake animals, who can actively tune their respiratory modulation. Still, we cannot exclude that sniffing properties affect oscillatory activities during odor sampling in the context of learning. A very recent paper showed that sniffing properties can be modulated by the context of the discrimination, i.e., which odor pair is presented during the test (Courtiol et al., 2014). However, the adjustment of sniffing parameters during odorant mixtures discrimination seems to rely largely on differences in sorption quality of the elements (Rojas-Líbano and Kay, 2012). Evolution of sniffing frequency and/or duration during the acquisition of the task is more likely to affect the intensity or the length of oscillations rather than its frequency.

**Figure 2:**
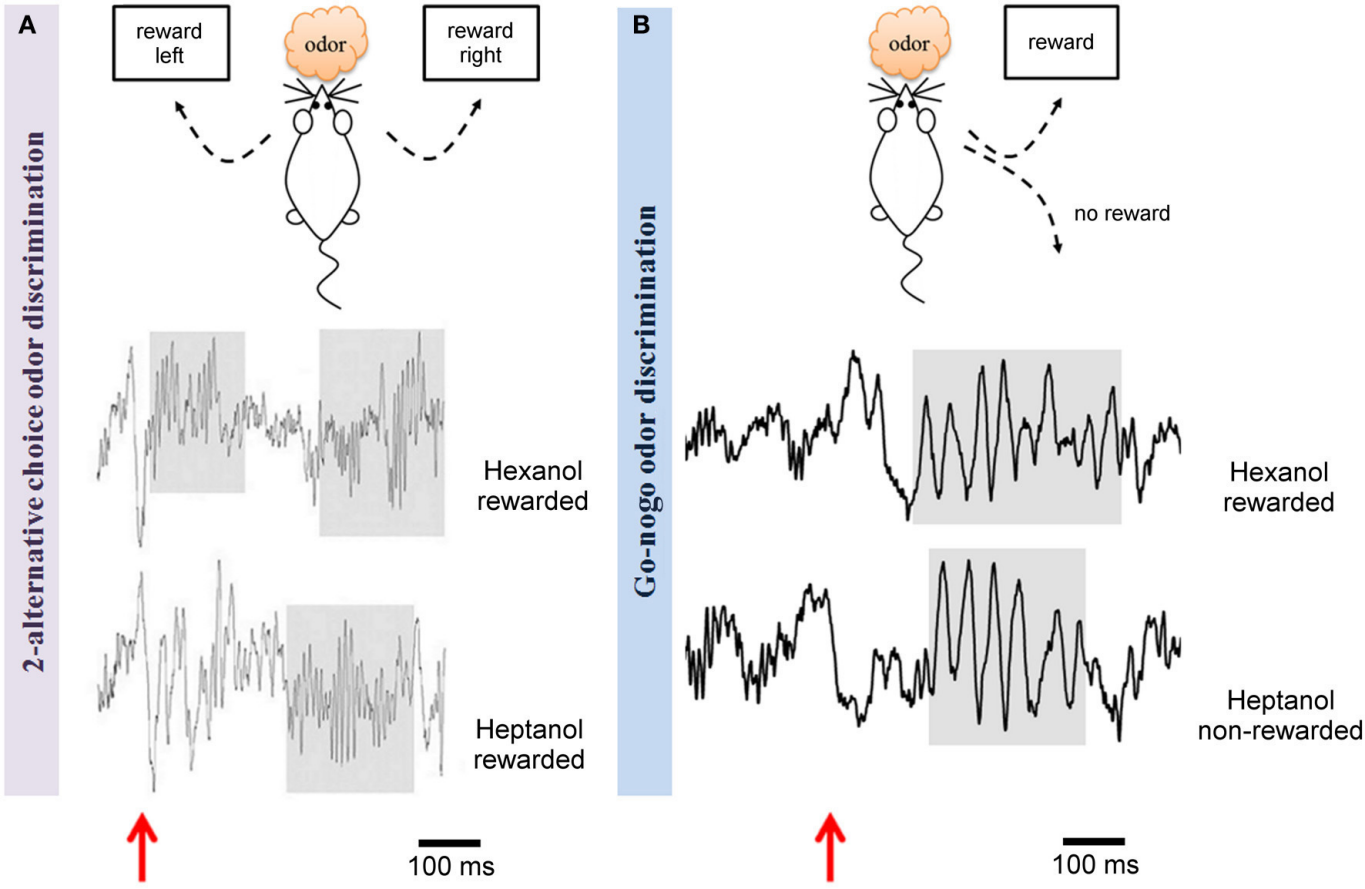
**Odor discrimination in two different behavioral tests led to distinct rhythms in the olfactory bulb**. Example of raw LFP traces recorded in the olfactory bulb during a criterion session of discrimination between two alcohols (hexanol and heptanol) in two different paradigms: **(A)** a two-alternative choice test (Beshel et al., 2007) or **(B)** a Go/No-Go test (Martin et al., 2007). The two tests are schematized on the top. The main difference is that both odors are rewarded in the 2 alternative-choice whereas one odor is not rewarded in the Go/No-Go. In both cases, the same odor concentration is delivered. Odorants are generated in glass test tubes by bubbling air (100 ml/min) through a column of pure odorant and injecting the odorized air into a carrier air stream (400 ml/min) via a computer-controlled olfactometer achieving ~20% saturated vapor. LFPs recorded during odorant sampling periods are displayed, red arrows pointed out odorant onset. As underlined by shaded regions, in the two-alternative choice test, odors evoke high gamma power (65–85 Hz) whereas in the Go/No-Go context, odorant sampling is associated with a strong beta wave (15–40 Hz). It has to be noted that both rewarded and unrewarded odors trigger beta rhythm in the Go/No-Go test.

We can rule out the hypothesis that only the concentration of odorant would turn beta into gamma in some conditions as it has been reported in anesthetized preparations (Neville and Haberly, 2003) and suggested in other studies (Rosero and Aylwin, 2011). Indeed, Go/No-Go and two-alternative choice tests were in this case performed in the same laboratory, using the same apparatus and the same odorant concentrations (Beshel et al., 2007; Martin et al., 2007). Beshel et al. (2007) show that the relatedness of the two odors involved in the discrimination increase gamma power in the MOB. However the relationship between the elevation of gamma power and the chemical proximity of the odorants, directly linked with the difficulty of the task, seems to be task specific. Indeed, as illustrated in Figure 2, the use of two chemically related odors heptanol and hexanol leads to different results in the two paradigms: beta band (15–40 Hz) power increase in the Go/No-Go paradigm (Martin et al., 2007) and enhanced gamma power (65–85 Hz) in the two-alternative choice task (Beshel et al., 2007).

How can we explain these discrepancies? One possibility could be that beta and gamma oscillations do correspond to distinct odor-related cognitive processing occurring at different stages of the training. Beta rhythm would be necessary, during the acquisition of the discrimination, to set up a broad network of distant brain structures required for specific rules and odor encoding. Indeed, one consistency across studies is that beta connects different brain areas (olfactory areas and beyond). Gamma could be required only in a subsequent stage of training, when odor discrimination has been learned by the rat, but its resolution is more challenging to reach the criterion. At this stage, the olfactory network has already been modified by learning and odor processing required for a fine sensory discrimination is rather supported by a local network and sustained by gamma band oscillations. This shift could allow a faster and more efficient treatment of odor, requiring less energy expenditure.

In the context of two-alternative choice task, beta rhythm is indeed present during the first learning sessions and disappears in subsequent sessions (Beshel et al., 2007; Kay and Beshel, 2010). In this condition, the number of trials required to reach the criterion for a given odor pair is enhanced compared to Go/No-Go for instance for the pair hexanol/heptanol, 350 trials (Beshel et al., 2007) vs. 72 trials (Martin et al., 2007) respectively (in both case, after rule transfer from previous odor pair discrimination). It is likely that the rat learns the discrimination between the odors during the first session, but that further sessions are necessary for the acquisition of the sensory-motor association. In the case where gamma oscillations are recorded in the context of a Go/No-Go task, the number of trials to criterion is also elevated (close to 1000) (Rosero and Aylwin, 2011).

The hypothesis that beta and gamma are two distinct mechanisms occurring at different time scale of the learning process is consistent with the idea proposed by Engel and Fries (2010) that beta band activity would dominate when top down input are the majority, whereas gamma band would rather reflects bottom-up local processing of sensory input. We will argue this hypothesis in the following part.

## Gamma and beta oscillations, local vs. distal networks?

In agreement with the notion that in brain circuits beta rhythms coordinate long-range communication whereas faster gamma rhythms are more related to local intra structure processing (Kopell et al., 2000; Siegel et al., 2012; Cannon et al., 2014), the emergence in the MOB and PCx of these two rhythms in odor-driven behavioral tasks is thus likely to sustain different network properties and processing.

In line with their implication in memory processes, beta oscillations have been found to sustain long range interactions. They have been recorded in many distant brain structures related to olfactory-driven behavior. Beyond the MOB and the PCx, they have been found in the lateral entorhinal cortex (Martin et al., 2004a; Igarashi et al., 2014), in the tubercle (Carlson et al., 2014), the hippocampus (Martin et al., 2007; Igarashi et al., 2014), in motor cortex M1 (Hermer-Vazquez et al., 2007) in different parts of the prefrontal cortex (infralimbic and orbito-frontal cortex), the basolateral amygdala, and the insular cortex (Chapuis et al., 2009). Interestingly, using an olfactory discrimination Go/No-Go task, van Wingerden et al. (2010) reported an increase in gamma oscillations in the orbitofrontal cortex, where power was correlated with rat training and performance, as shown by Beshel et al. (2007) in the MOB. However, in the same study, the authors also observe some late beta oscillatory activity more associated with odorant sampling and very similar to what was reported in the MOB or the hippocampus (Martin et al., 2004b, 2007). This shift from gamma to beta rhythm observed in several areas associated with odorant sampling just before the animal makes a decision is in agreement with the hypothesis of a general beta synchronization across odor-processing areas that could be the signature of a functional network set up through learning.

Whereas gamma oscillations recorded in these structures are likely to reflect local processing and thus to have a distinct origin from that recorded in primary olfactory structures, we propose that beyond the MOB and PCx, beta oscillations would tag brain structures involved in the behavioral task that the animal is performing and form a unique representation of the odor in this task. Studies where multielectrode recordings have been performed have shown that beta increase occurred specifically in brain regions involved in the task performed by the animal. Indeed, in Martin et al. (2007), beta does not increase in the hippocampus for the first odor discrimination but for the transfer that is more likely to involve the structure. In the same way, after odor aversive conditioning, beta oscillations increase in insular and infralimbic cortices when the odor is ingested but not when it is delivered by airflow (Chapuis et al., 2009). By extension, we postulate that other brain areas, not yet studied, are capable of joining beta oscillatory network if involved in a given olfactory task.

One striking and stable characteristic of the emergence of beta rhythm in olfactory structures is that it is narrowly linked to behavioral output. Interestingly, beta power modulations seem to follow some aspects of the learning curve dynamics at least in the MOB and PCx. Indeed, beta gradually increases across training sessions (Martin et al., 2004b) and a strong beta oscillatory activity is observed just one or two sessions before the learning criterion was reached regardless of the time needed by the rat to acquire the discrimination (Ravel et al., 2003; Martin et al., 2004b, 2007). This is also true when odor learning is achieved in 1 day in the context of aversive odor learning (Chapuis et al., 2009) or following rule transfer to a new odor pair discrimination, which is done within one learning session (Martin et al., 2007). It is important to underline that beta power increases specifically for the learnt odor pair and falls down at the beginning of each new odor pair presentation. If training is continued post-criterion, beta power decreases as a function of overtraining (Martin et al., 2007). On the contrary, we observe that once the discrimination is achieved, if animals are put at rest and not tested for a long period (from a week to a month), beta oscillation emerges again stronger than ever. This latter effect is observed both after appetitive and aversive conditioning (Martin et al., 2004b; Chapuis et al., 2009). Finally, the emergence of beta oscillatory activity in a network seems to be highly specific of the conditioning procedure. Taking advantage of two different experimental situations suitable to induce a conditioned olfactory aversion we were able to demonstrate two different odor-evoked beta networks according to how the odor has been previously experienced by the animal (Chapuis et al., 2009).

All together these observations have therefore spawned the idea that beta rhythm might be necessary to bind together elements of a broad network and contribute to the build-up of memory. Indeed, we make the hypothesis that such a coordinated oscillatory activity could be used to tag preferentially inter or intra area connections that need to be reinforced to be efficiently and rapidly reactivated when the odor is further encountered. This idea has been strongly reinforced by a recent article that identified beta oscillations coupling between the entorhinal cortex and the hippocampus as a mechanism for the emergence of a functional circuit during encoding of odor associative memory (Igarashi et al., 2014).

**Figure 3:**
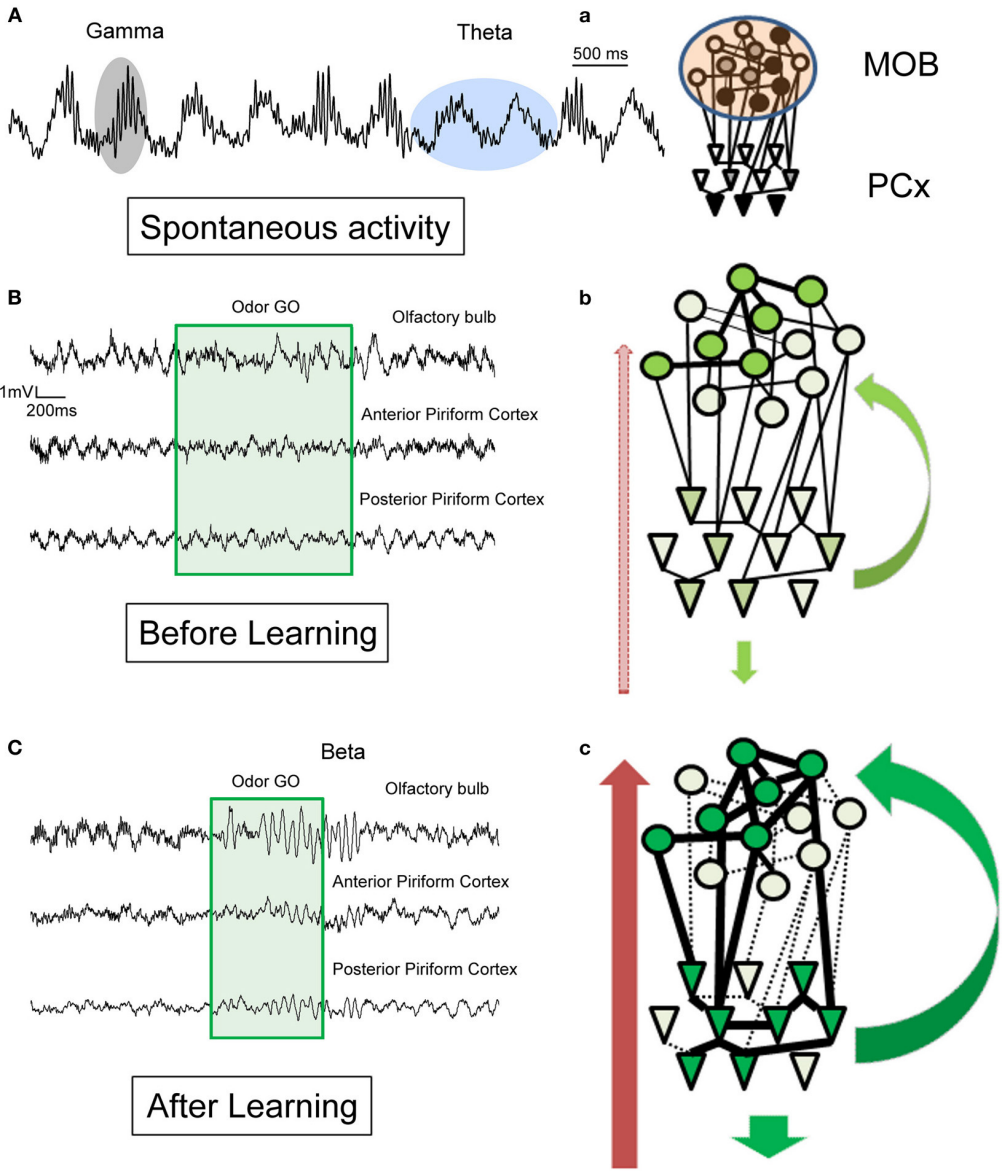
**Schematic illustration of hypotheses for the generation of beta (15–35 Hz) and gamma (60–90 Hz) rhythms in the olfactory bulb and the piriform cortex in awake behaving animals**. Example of raw LFP traces recorded **(A)** in the MOB in the absence of olfactory stimulation and **(B)** in MOB and two different regions of the PCx before learning and **(C)** after learning during a conditioned discrimination paradigm (Go/No-Go). **a–c**: Corresponding schematic representation of MOB and PCx interconnected networks and centrifugal modulation. The level of neuromodulation is represented by the red arrow on the left, the level of cortical feedback by green arrow on the right. **(A)** Spontaneous activity: in the absence of olfactory stimulation. Observe the regular theta respiratory modulation (around 2 Hz) and the associated bursts of gamma activity (60–90 Hz). See also in **(B)** and **(C)** the portion just preceding the odorant sampling (green square area) how the gamma bursts decrease in the posterior part of the PCx compare to the MOB and anterior part of PCx. **a**: In the absence of olfactory stimulation, the level of activation in both networks is weak and variable and both structures are dominated by theta and gamma activity. Gamma activity is transmitted from the MOB to the PCx. **(B)** Before learning: during odorant sampling, occurrence of gamma bursts is reduced but recovered after the animal has left the odor port. **b**: During this phase, a population of mitral cells of the MOB becomes active, this input is transmitted to a corresponding population of pyramidal cells. Both neuromodulatory and cortical feedback are exerted on the networks. However, no real coordination is set up in the network. **(C)** During training: In addition to a strong and sustained decrease in gamma activity, a clear beta oscillation is observed in the MOB and two regions of PCx associated to odorant sampling. **c**: During this phase, we propose that both assemblies of active mitral cells and pyramidal cells reinforce their connections. The result could be a more efficient and rapid transfer of olfactory information. This coordination is under the influence of both cortical feedback and neuromodulatory fibers as suggested by the results we observed with lidocaine inactivation of the peduncle (Martin et al., 2006). Once synaptic contacts are established, if the training is maintained to get over training, the amplitude of beta oscillatory activity decreases. On the contrary, if the animal is left in his home cage for a long interval without training and tested again, both structures exhibit a very strong beta oscillatory activity.

The shift between gamma and beta when an odor is processed is likely due to a change in bidirectional connections between the MOB and other cerebral structures (Figure 3).

It has been reported for a long time that centrifugal influences are gating synaptic plasticity processes in both MOB and PCx (for a review see Mandairon and Linster, 2009). As expected, manipulations of centrifugal projections alter behavior. The lesion of efferent inputs to the MOB prevents the formation of odor-reward associations, but has no effect on the resolution of spontaneous habituation experiment (Kiselycznyk et al., 2006). Besides, specific manipulation of noradrenergic action to the MOB impairs mice in discrimination learning in a Go/No-Go paradigm (Doucette et al., 2007). In this same task, MT cells undergo a profound change in odor responsiveness throughout a learning session (Doucette and Restrepo, 2008), that is dependent on centrifugal feedback (Restrepo et al., 2009). Both cortical feedback and neuromodulatory influences play a determinant role in the shift between gamma and beta rhythms as indicated by the impact of their local blockade on the odor-evoked activity in both the MOB and the PCx in a Go/No-Go task (Martin et al., 2006), reinforcing their link with expression of plasticity and memory processes. Interestingly, directed coherence analyses have shown that during odorant sampling, the MOB would drive odor related beta activity to the PCx (Boeijinga and Lopes da Silva, 1989; Kay and Beshel, 2010) and to the hippocampus (Chapman et al., 1998; Gourévitch et al., 2010), carrying relevant information in the bottom up direction instead of the reverse. In contrast, it could be the opposite during memory consolidation since in a slow-wave sleep-like state induced under anesthesia, the functional link based on slow waves LFP recordings (<15 Hz) is in the direction of the hippocampus to the PCx (Wilson and Yan, 2010). That beta rhythm could play a role in memory consolidation during sleep remains an open question.

The ultimate argument for a causal link between emergence of oscillatory activities and improvement of behavioral performance would be to degrade beta/gamma oscillatory dynamic in the network and observe behavioral impairment. Few studies have been conducted in MOB mammals that mainly addressed gamma activity. Local injection of low doses of picrotoxin, a GABAa receptor antagonist were reported to enhance gamma oscillations and also led to behavioral modifications: mice displayed an increased odorant sampling time, and their performances were selectively altered in the case of a fine odor discrimination in a Go/No-Go paradigm (Lepousez and Lledo, 2013). Nusser et al. (2001) used a transgenic mouse model, in which GABAa receptors were disrupted specifically on granule cells, i.e., those cells where centrifugal feedback targets and reported gamma oscillations power was enhanced in the MOB. Behavioral testing concluded that whereas mice seemed to perform better on a simple odor identification task, they were impaired on a mixture discrimination test. Interestingly, these two studies show that network modifications that lead to gamma band increase also result in behavioral impairment. Using pharmacological blockade, we have reported that inactivation of feedback projections abolish beta oscillations and conversely increases gamma power (Martin et al., 2006). Consequently, we postulate that those modifications that increase gamma oscillations and also lead to beta weakening impair behavior.

## Conclusion

In this review, we focused on two different oscillatory rhythms beta (15–40 Hz) and gamma (60–100 Hz) that have been associated with olfactory stimulus processing. We propose that gamma activity is associated with the resting state of a network limited to the first two steps of the olfactory system (MOB and anterior part of the PCx). As reported, this basic activity could be modulated in power during learning according to some experimental conditions but rather reflects the involvement of a limited local network under the control of higher cortical feedback and neuromodulators. In behaving animals, as soon as an odor is processed, this local coordination is disrupted and replaced by a lower frequency oscillation in the beta range (15–40 Hz). Most of the data reported in this review lead to the hypothesis that beta activity is the signature of a larger network including not only olfactory sensory areas but also each structure involved in the processing of the odorant stimulus, which could differ according to the behavioral situation. As stated in the present article, a decent amount of data is in favor of a strong correlation between beta oscillation modulation in power and learning-induced changes, in both rats and mice. Beta rhythm frequency is well suited for long range interactions (Kopell et al., 2000; Von Stein and Sarnthein, 2000) and thus for sustaining memory processes. The presence of beta oscillatory rhythm within and between neuronal networks would optimize information processing, representing a framework for neuronal synchronization. By this mean odor coding would be more efficient and temporal simultaneity would favor hebbian mechanism of plasticity (Cassenaer and Laurent, 2007). However, we still lack evidence to disambiguate whether beta oscillations are instrumental for processes like spike timing plasticity in the network or if on the contrary they are just reflecting these changes. Nevertheless, we propose the idea that mapping such oscillatory activities in a neural network could be a good way to assess learning-induced brain plasticity at least in the context of odor-guided tasks. Recently, beta oscillations have also been used as a tool to reveal impaired network activity preceding behavioral dysfunctions (Wesson et al., 2011) and evaluate the impact of a treatment to enhance clearance of beta-amyloid protein in a mouse model of Alzheimer disease (Cramer et al., 2012). Providing experimental evidence to support a causal link between oscillatory binding and inter area synchronization will be one of the main goal for the future.

### Conflict of interest statement

The authors declare that the research was conducted in the absence of any commercial or financial relationships that could be construed as a potential conflict of interest.
